# Hemoglobin-to-red blood cell distribution width ratio in the relationship between occupational aluminum exposure and cognitive function impairment: a mediation analysis

**DOI:** 10.3389/fpubh.2025.1639229

**Published:** 2025-09-18

**Authors:** Ruo-nan Wang, Zhuo-hui Wang, Wen-cheng Hu, Fan-peng Kong, Zi-yan Pei, Jing Song, Xiao-ting Lu, Bao-long Pan

**Affiliations:** ^1^Department of Occupational Health, School of Public Health, Shanxi Medical University, Taiyuan, Shanxi, China; ^2^Sixth Hospital of Shanxi Medical University (General Hospital of Tisco), Taiyuan, Shanxi, China

**Keywords:** occupational aluminum exposure, cognitive function, blood indicators, inflammatory markers, mediating analysis

## Abstract

**Objective:**

To investigate the partial mediating role of the inflammatory marker hemoglobin-to-red cell distribution width ratio (HRR) in the association between occupational aluminum exposure and cognitive function impairment, and its significance.

**Methods:**

In this study, 401 workers from a Shanxi aluminum plant were selected by Cluster Sampling. Fasting elbow venous blood was collected for measuring routine blood counts, plasma aluminum concentration (P-Al) was measured using inductively coupled plasma mass spectrometry (ICP-MS), Montreal Cognitive Assessment (MoCA) was used to assess the cognitive function. Multiple linear regression was used to analyze the relationship between P-Al and cognitive function and HRR, and a restricted cubic spline model was used to fit the dose–response relationship, and mediated effects analysis was performed.

**Results:**

The median plasma aluminum concentration ( $P_{25},P_{75}$ ) of the workers was 50.74 (23.45, 85.52) μg/L, the mean HRR was 11.87, the median MoCA total score ( $P_{25},P_{75}$ ) was 24.00 (22.00, 26.00). A dose–response relationship showed that the MoCA score decreased with increasing P-Al. After adjusting for demographic and lifestyle covariates, multiple linear regression showed that P-Al was negatively correlated with the HRR and MoCA score. For each unit increase in P-Al, the HRR decreased by an average of 0.17, and the total MoCA score decreased by an average of 0.9. HRR mediated 9.89% of the effect between P-Al and MoCA score.

**Conclusion:**

Occupational aluminum exposure negatively affects workers’ cognitive function and HRR levels. HRR can partially explain the effects of occupational aluminum exposure on workers’ cognitive function.

## 1 Introduction

Aluminum, the most abundant metallic element in the Earth’s crust (about 8.88%), is widely used in industries like aerospace, transportation, machinery, and power due to its high conductivity, processability, and plasticity. China, the world’s largest aluminum producer and consumer, has seen extensive occupational aluminum exposure from large-scale electrolytic aluminum production. Numerous studies have established an association between occupational aluminum exposure and cognitive impairment in workers (1).

Accumulating evidence indicates that aluminum enters the human body primarily through the respiratory and digestive tracts, exerting toxic effects on the nervous, skeletal, endocrine, and hematopoietic systems (2). Specifically, its hematotoxin effects frequently manifest as non-iron deficiency anemia and renal anemia (3). Mechanistically, aluminum disrupts erythropoiesis, resulting in morphological abnormalities (including acanthocytes and target cells) and elevated erythrocyte heterogeneity (4). Consistent with these findings, a study demonstrated significant reductions in hemoglobin levels, erythrocyte counts, and hematocrit values in aluminum-exposed rats (5). Collectively, these findings strongly suggest that aluminum and its compounds significantly impact the hematopoietic system.

Conventional blood indicators in the hematopoietic system include white blood cell count (WBC), lymphocyte count, neutrophil count, monocyte count, red blood cell count (RBC), and hemoglobin (HGB). Mean corpuscular volume (MCV), mean corpuscular hemoglobin content (MCH), mean corpuscular hemoglobin concentration (MCHC), hematocrit (PCV), platelet count (PLT), and red blood cell distribution width (RDW), etc. (6). Studies have found that lower MCH and RDW are associated with relatively lower language, numerical reasoning, and numerical memory, and lower hemoglobin levels in the blood are linked to poor cognitive function and AD (7). The comprehensive inflammation index is easy to measure and cost-effective, and has been widely applied in clinical and research fields. Studies have shown that there is a correlation between the level of immune cells in peripheral blood and the risk of AD (8). A study exploring the correlation between blood inflammatory indicators and the severity of cognitive impairment in patients with Alzheimer’s disease indicated that the neutrophil/lymphocyte ratio (NLR), monocyte/lymphocyte ratio (MLR), and platelet/lymphocyte ratio (PLR) have become important independent risk factors for AD, NLR is regarded as having the greatest potential as a biomarker in AD (9). It is reported that neuroinflammation plays a significant role in promoting neurodegeneration in humans. *In vivo* studies have shown that inflammatory cells are activated in patients with AD, other dementias, and MCI (10). A significant portion of recent biomarker research has focused on peripheral inflammatory markers, which are key elements of neurodegeneration, including various cytokines, lymphocytes, neutrophils, other blood cells, and their counts and related ratios (11). Dysregulation of neuroinflammatory responses has been well documented in AD and MCI.

Hemoglobin (Hb), an oxygen-carrying protein predominantly found in red blood cells, serves as a key component of complete blood count analysis. Hb levels correlate with nutritional status and immune function, providing an indicator of anemia severity. Emerging evidence suggests Hb may also function as a marker of chronic inflammation (12). RDW, a measure of erythrocyte size heterogeneity, has been identified as an inflammatory marker. Under inflammatory conditions, erythrocyte lifespan is reduced, resulting in anemia and elevated RDW (13). Elevated levels of RDW are associated with increased risks of diabetes, strokes, and chronic health conditions, as well as higher overall mortality. Notably, a study demonstrated a J-shaped association between RDW and dementia risks in older adults (14).

The hemoglobin-to-red cell distribution width ratio (HRR), calculated as Hb/RDW, represents a novel inflammatory marker proposed by Sun et al., which can effectively reflect the levels of oxidative stress and systemic inflammatory response in the body (15). Although HRR was first reported in 2016, its application value in various disease fields has only gradually attracted widespread attention in recent years. A study on HRR and stroke has shown that inflammation can disrupt iron metabolism, leading to a decrease in hemoglobin levels and an increase in red blood cell heterogeneity, thereby reducing the Hb/RDW ratio. HRR is negatively correlated with stroke. The higher the HRR, the lower the risk of stroke (16). Previous studies have found that people with lower hemoglobin levels have more severe cognitive decline, an increased risk of dementia, and elevated RDW is also associated with an increased risk of AD (17). Another study on the relationship between HRR and cognitive dysfunction after stroke found that patients with decreased HRR levels had an increased risk of cognitive dysfunction, and the two showed a negative linear relationship (18), and in a cross-sectional study on the ratio of hemoglobin to red blood cell distribution width and depression in older adults, it was shown that HRR may be an independent risk factor for depression, being more powerful than Hb or RDW alone. It may help detect depression early, prevent clinical deterioration and recurrence, and can also serve as a therapeutic target (19). Inflammation constitutes a critical mechanism underlying cognitive dysfunction. Extensive scientific evidence indicates that dysregulation of this physiological response to tissue damage is not properly controlled, a chronic, low-grade state of inflammation occurs, and that this inflammation plays a key role in the pathogenesis of cognitive decline and dementia (20). To overcome single-marker limitations, we used a blood-based inflammatory composite score to evaluate its link to aluminum-induced cognitive decline.

We thus hypothesize that HRR mediates aluminum-induced cognitive impairment. Current studies largely focus on aluminum’s impact on the overall blood system, with limited research on its relationship with inflammatory markers and cognitive function. Occupational aluminum workers, due to their unique exposure, are more vulnerable to harmful effects. This study thus examines the relationship between plasma aluminum concentration, HRR level, and cognitive function in these workers.

## 2 Objects and methods

### 2.1 Study population

This study adopts the method of cluster sampling, mainly selecting on-the-job male workers in the electrolytic aluminum workshop and alumina workshop in a large-scale aluminum plant in Shanxi in 2019. Its leading products are alumina and aluminum hydroxide, and the production process adopts the more mature mixed-linkage method in China. We recruited 434 male workers and excluded 33 subjects based on the following criteria: service <1 year (*n* = 5); long-term use of aluminum-containing medications or foods (*n* = 4); diseases affecting blood routine indexes (such as anemia, hematological or cardiovascular disorders; *n* = 7); genetic mental or neurological disorders in the subject or family members (*n* = 2); extremely uncooperative subjects (*n* = 2); and missing blood data or plasma aluminum concentration data (*n* = 13). Ultimately, 401 participants were included. Data were collected via a standardized occupational health questionnaire administered through face-to-face interviews by trained investigators. The survey covered demographics, lifestyle habits, medical history, and occupational experience, and cognitive function was assessed in a quiet environment. All participants provided written informed consent, and the study was approved by the Medical Ethics Committee of Shanxi Medical University.

### 2.2 Measurement of plasma aluminum concentration

Using sodium heparin anticoagulation tubes, 2 mL of whole blood was collected from workers in the early morning fasting state and centrifuged at 1000 r/min (*r* = 12 cm) for 10 min, and the upper layer of separated plasma was pipetted into a 1.5 mL centrifuge tube. 400 μL of plasma was added to 1.6 mL of diluent containing 0.1% Triton and 4% nitric acid and mixed thoroughly, and left at room temperature for 24 h. The supernatant was then centrifuged at 12,000 r/min (*r* = 6.5 cm) for 15 min for detection. The aluminum concentration in plasma was determined by inductively coupled plasma mass spectrometry (NexlON 300D, PerkinElmer, United States) (21). All laboratory utensils were made of plastic, and glass was not allowed to be used to prevent the aluminum contained in the utensils from affecting the results of the assay.

### 2.3 Routine blood measurements

Sodium heparin anticoagulant tubes were used to collect 5.0 mL/person of fasting elbow venous blood from workers in the early morning, which was gently mixed at room temperature using a Sysmex XN1000 Hematology Analyzer, and the test was completed within 2 h. Routine blood measurements included RBC, Hb, RDW, WBC, and PLT. Instrument calibration is performed before testing, and quality control products are run daily to ensure that the coefficient of variation (CV) within a batch is <5%.

### 2.4 HRR calculations

Calculate the hemoglobin to red blood cell distribution width ratio (HRR) by dividing the hemoglobin (HGB) in grams per liter (g/L) by the red blood cell distribution width (RDW), with the detection unit being ‘%’. The normal reference value range for each indicator: HGB (120.00–160.00); RDW (11.50–14.50), any value below or above the normal reference range is defined as abnormal for this indicator. For accuracy, the result is rounded to two decimal places.

### 2.5 Cognitive assessment

In this study, we used the Montreal Cognitive Assessment (MoCA) to assess the cognitive function of workers. It was administered by professional nurses from the Department of Neurology of the First Hospital of Shanxi Medical University in a standardized and quiet environment in a one-to-one manner. The MoCA scale covers a wide range of cognitive domains and has high sensitivity and specificity for the identification of MCI (22). The scale has a total score of 30; a score of <26 points suggests the occurrence of cognitive impairment, and one point will be added to the score if the participant has less than 12 years of education.

### 2.6 Other variables

In this study, the Body Mass Index (BMI) is calculated by dividing an individual’s weight in kilograms (kg) by the square of their height in meters ( $m^{2}$ ); The calculation method for per capita monthly household income is the total monthly household income divided by the total number of household members, it is classified into three categories: <1999, 2000–4,999, and >5,000. According to the per capita disposable income of residents in the research area, they correspond, respectively, to the low, middle, and high-income groups; smoking was categorized into non-smokers and smokers (defined as those who smoked ≥1 cigarette/day for ≥6 months); alcohol consumption into non-drinkers and drinkers (defined as those who drank alcohol ≥1 time/week for ≥6 months); educational level was categorized into junior high school and below and high school and above; exercise into non-exercisers and exercisers (defined as those who engaged in ≥3 sessions/week of moderate-to-high intensity activities lasting ≥30 min each); marital status into married (including married, divorced, and widowed) and unmarried; and years of service were calculated from the time workers entered the aluminum plant to the start of this survey.

### 2.7 Statistical analysis

R 4.3.0 statistical analysis software was used in this study, all the research subjects were divided into the low-aluminum exposure group and the high-aluminum exposure group according to the median plasma aluminum concentration Normal distribution of continuous data was expressed as mean± standard deviation (Mean ± SD), and comparisons between multiple groups were performed using analysis of variance (ANOVA); Skewed distribution of continuous data was expressed as median and interquartile spacing M ( $P_{25,}P_{75}$ ), and comparisons between groups were performed using the Kruskal-Wallis H rank sum test; categorical data were expressed as N (%), and comparisons between groups were made using the chi-square test or Fisher’s exact probability method. *p* < 0.05 was considered a statistically significant difference. To improve data normality, plasma aluminum concentrations were natural log-transformed, and raw values for Hb, RDW, and cognitive scores were used for subsequent analyses.

Multiple linear regression models were used to explore the relationship between P-Al, HRR, and MoCA total scores, and univariate analyses were not adjusted for confounding variables, such as age, BMI, duration employment, education level, per capita monthly household income, smoking, alcohol consumption, exercise, and marital status, so further multivariate analyses were performed to rule out the above variables, followed by restricted cubic spline (RCS) to reflect the dose–response relationship of P-Al with HRR and MoCA total score.

RCS indicates the existence of nonlinear correlations that can assist in identifying potential threshold intervals. Threshold analysis is conducted using the ‘segmented’ package. The threshold effect refers to the phenomenon where the magnitude of the effect on the dependent variable changes when the independent variable exceeds a certain point. Through threshold effect analysis, important inflection points that significantly affect the correlation between variables can be identified. In the threshold analysis results, Model 1 is a regular regression analysis, while Model 2 is a two-stage regression. By observing the *p*-values above and below the inflection point, the correlation between exposure and outcome is determined. Here, “effect” represents the effect value in the regression; The *P* for the likelihood test is a likelihood ratio test. *p* > 0.05 indicates that there is no significant improvement in Model 1 compared with Model 2. If *p* < 0.05, it suggests that there is a significant difference in the association strength before and after the inflection point, and the inflection point is statistically significant.

Mediation analysis examines how the effect of an exposure variable on an outcome variable is mediated through a third variable, given the association between the independent and dependent variables. The mediating effect requires three conditions (23): (1) the exposure variable significantly affects the outcome variable; (2) the exposure variable significantly affects the mediating variable; and (3) the mediating variable significantly affects the outcome variable. In this study, the ‘mediation’ package of R language was used to assess HRR’s mediating role in cognitive function changes due to aluminum exposure, with significance assessed at *α* = 0.05 using two-sided tests.

## 3 Results

### 3.1 General characteristics of participants

A total of 401 male workers participated in this study. The age of the study participants M ( $P_{0},P_{100}$ ) was 45.0 (23.0, 57.0) years, the length of service M ( $P_{0},P_{100}$ ) was 25.0 (3.0, 39.0) years, the average per capita monthly income of the family was mostly in the range of 2000–4,999 yuan (83.54%), most of them were alcohol drinkers (50.37%), and more than half of the male workers smoked cigarettes (66.33%). The percentage of infrequent exercisers was higher (56.86%), and marital status was mainly married (96.76%). Table 1 summarizes the general demographic characteristics grouped by blood aluminum concentration, there were statistically significant differences between the two groups in terms of age, length of service, exercise status, plasma aluminum level, HRR ratio and total MoCA score (*p* < 0.05), while the differences in BMI, education level, per capita monthly household income status, smoking, alcohol consumption, and marital status were not statistically significant (*p* > 0.05).

**Table 1:** General demographic characteristics.

Variables	Total (*n* = 401)	Low-aluminum exposure group (*n* = 200)	High-aluminum exposure group (*n* = 201)	*p*
P-Al, (μg/L)	50.74 (23.45, 85.52)	23.31 (10.48, 36.02)	85.52 (68.10, 120.99)	**<0.001**
Age, (years)	45.00 (39.00, 49.00)	47.00 (41.00, 49.25)	44.00 (38.00, 49.00)	**0.009**
BMI, ( $\mathrm{kg}/m^{2}$ )	24.22 (21.22, 26.93)	24.55 (21.61, 27.02)	23.45 (20.98, 26.60)	0.081
Duration employment (years)	25.00 (17.00, 29.00)	26.00 (20.00, 30.00)	22.00 (14.00, 29.00)	**0.008**
Sum of MoCA	24.00 (22.00, 26.00)	24.00 (22.00, 26.00)	23.00 (21.00, 25.00)	**0.025**
HRR	11.87 ± 0.88	11.99 ± 0.81	11.75 ± 0.93	**0.007**
*Per Capita* Monthly Household Income (RMB)				0.189
<1999	51 (12.72)	30 (15.00)	21 (10.45)	
2000–4,999	335 (83.54)	165 (82.50)	170 (84.58)	
>5,000	15 (3.74)	5 (2.50)	10 (4.98)	
Education level				0.883
Junior high school and below	210 (52.37)	104 (52.00)	106 (52.74)	
High school and above	191 (47.63)	96 (48.00)	95 (47.26)	
Smoking				0.159
No	135 (33.67)	74 (37.00)	61 (30.35)	
Yes	266 (66.33)	126 (63.00)	140 (69.65)	
Drinking				0.099
No	199 (49.63)	91 (45.50)	108 (53.73)	
Yes	202 (50.37)	109 (54.50)	93 (46.27)	
Exercise				**0.031**
No	228 (56.86)	103 (51.50)	125 (62.19)	
Yes	173 (43.14)	97 (48.50)	76 (37.81)	
Marriage				0.771
No	13 (3.24)	7 (3.50)	6 (2.99)	
Yes	388 (96.76)	193 (96.50)	195 (97.01)	

Note: Bolded *p*-values indicate statistically significant differences between groups (*p* < 0.05).

P-Al, plasma aluminum; BMI, Body Mass Index; HRR, hemoglobin-to-red cell distribution width ratio; the measurement data are Mean ± SD or M (*P*_25_, *P*_75_), and the counting data are expressed as N (%).

### 3.2 Relationship between aluminum exposure and cognitive scores

With the help of a multiple linear regression model, the effect of aluminum exposure level on cognitive scores was deeply analyzed, and the results of the analysis showed that there was a significant negative correlation between the aluminum exposure level and the total MoCA scores under the dual validation of univariate and multivariate analyses. As shown in Table 2, for each unit increase in aluminum exposure level, the MoCA total score was reduced by 0.89 points on average [*β* = −0.89, 95% CI (−1.50–−0.27)], and this negative correlation remained significant even after controlling for other variables [*β* = −0.90, 95% CI (−1.50–−0.30)].

**Table 2:** Relationship between plasma aluminum levels and cognitive scores.

Variables	Single factor		Multifactorial	
	β (95% CI)	*p*	β (95% CI)	*p*
P-Al	−0.89 (−1.50–−0.27)	**0.005**	−0.90 (−1.50–−0.30)	**0.004**
Age	−0.09 (−0.14–−0.05)	**<0.001**	−0.07 (−0.15–0.00)	0.053
BMI	0.07 (−0.00–0.15)	0.051	0.04 (−0.03–0.11)	0.268
Duration employment	−0.07 (−0.10–−0.03)	**<0.001**	−0.01 (−0.07–0.06)	0.867
*Per Capita* Monthly Household Income				
<1999	Reference		Reference	
2000–4,999	0.13 (−0.82–1.08)	0.791	0.02 (−0.88–0.92)	0.967
>5,000	0.59 (−1.26–2.45)	0.532	−0.01 (−1.77–1.75)	0.989
Education level				
Junior middle school and below	Reference		Reference	
High school and above	1.80 (1.19–2.40)	**<0.001**	1.38 (0.75–2.01)	**<0.001**
Smoking				
No	Reference		Reference	
Yes	−0.04 (−0.71–0.63)	0.910	−0.14 (−0.79–0.52)	0.685
Drinking				
No	Reference		Reference	
Yes	0.84 (0.22–1.47)	**0.009**	0.80 (0.19–1.41)	**0.010**
Exercise				
No	Reference		Reference	
Yes	0.65 (0.02–1.28)	**0.044**	0.38 (−0.23–1.00)	0.221
Marriage				
No	Reference		Reference	
Yes	0.02 (−1.76–1.80)	0.982	0.40 (−1.28–2.09)	0.638

Note: Bolded *p*-values indicate that the association between the variable and cognitive scores is statistically significant (*p* < 0.05).

Adjusted age, BMI, duration employment, education level, per capita monthly household income, smoking, alcohol consumption, exercise, and marital status. P-Al, plasma aluminum; BMI, Body Mass Index.

**Figure 1:**
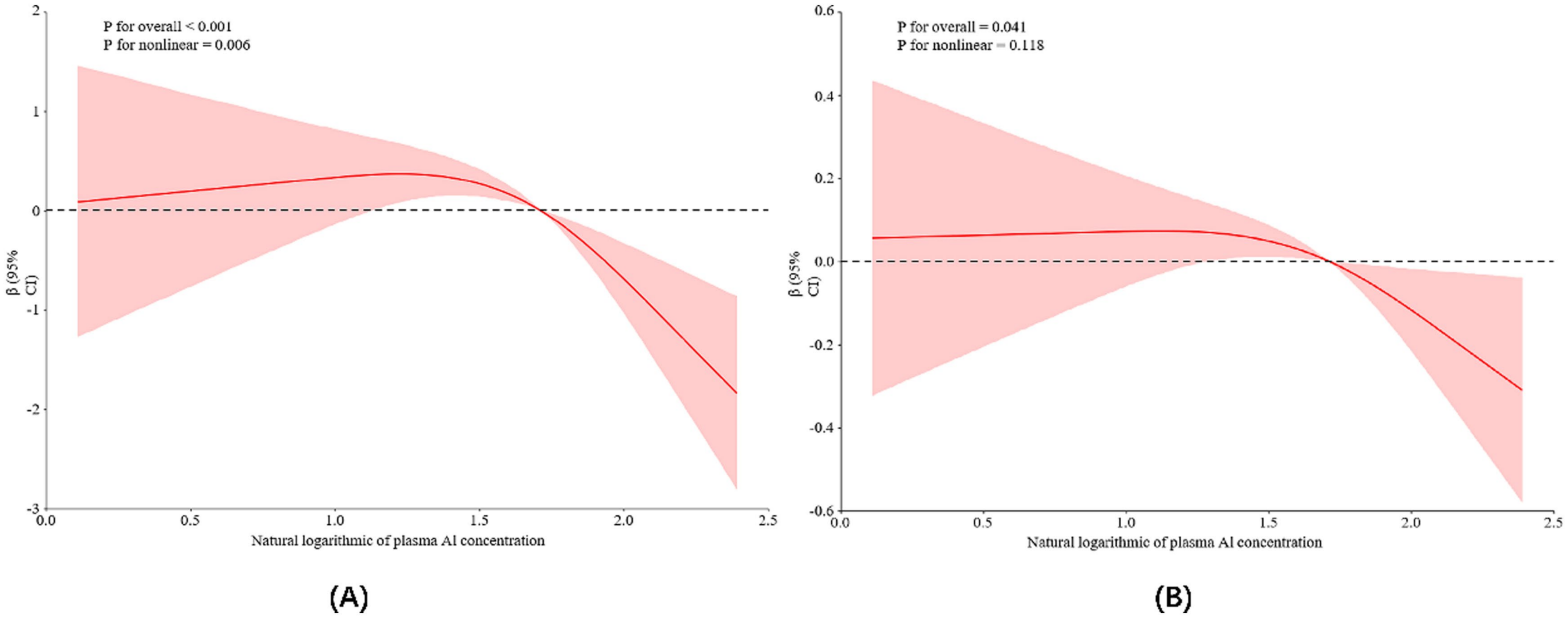
Restricted cubic spline model representing the association between log-transformed plasma aluminum concentrations and cognitive scores **(A)** and HRR levels **(B)**. The mentioned cognitive scores and HRR levels are adjusted for age, BMI, length of service, per capita monthly household income, smoking and alcohol consumption, education level, exercise, and marital status. Red curves: indicate the association between plasma aluminum concentration and cognitive scores or HRR levels; pink shaded areas: indicate 95% confidence intervals for *β* values.

In addition, the RCS model confirmed the dose-effect relationship between plasma aluminum concentration and total MoCA score, as shown in Figure 1A. A nonlinear relationship between aluminum exposure and total MoCA score could be seen based on the RCS curves, and a threshold effect analysis showed that there was a threshold effect between the level of aluminum exposure and total MoCA score (*p* = 0.002). As seen in Table 3, when the natural logarithmic value of blood aluminum concentration was lower than 1.81, no significant association was found with the MoCA score; when it was higher than 1.81, the aluminum exposure level was negatively associated with the MoCA score [*β* = −3.85, 95% CI (−6.42–1.28)], suggesting that the association between aluminum exposure and cognitive impairment was significantly strengthened when the plasma aluminum concentration was above the threshold, and this threshold may reflect the critical point where aluminum breaks through the physiological defense mechanism and triggers neurotoxicity. This finding provides an important basis for the development of safety standards for aluminum exposure and early intervention.

**Table 3:** Threshold analysis between plasma aluminum levels and total MoCA score.

Sum of MoCA	Effect	*p*
Model 1 Fitting model by standard linear regression	−0.90 (−1.50–−0.30)	**0.004**
Model 2 Fitting model by two-piecewise linear regression		
Inflection point	1.81	
<1.81	0.02 (−0.88–0.91)	0.973
≥1.81	−4.17 (−6.77–−1.57)	**0.002**
P for likelihood test		**0.002**

Note: The bolded *p* value indicates a significant correlation between plasma aluminum levels and the total MoCA score.

Model 1, Model 2 adjusted age, BMI, duration employment, education level, per capita monthly household income, smoking, alcohol consumption, exercise, and marital status.

### 3.3 Relationship between aluminum exposure level and HRR

Analyzing the relationship between aluminum exposure level and HRR, the results of univariate analysis showed that for each unit increase in aluminum exposure level, HRR decreased by 0.21 units on average (*p* < 0.05), and after controlling for the other variables, this relationship was still significant, but with a slight decrease in *β*-value (from −0.21 to −0.17), as shown in Table 4. Based on the RCS curves as well as the analysis of the threshold effect, it was found that there was a dose–response relationship between HRR levels and plasma aluminum concentrations, but no nonlinear relationship was found between the two (As shown in Figure 1B; Table 5).

**Table 4:** Relationship between plasma aluminum levels and HRR.

Variables	Single factor		Multifactorial	
	β (95% CI)	*p*	β (95% CI)	*p*
P-Al	−0.21 (−0.38–−0.04)	**0.014**	−0.17 (−0.34–−0.01)	**0.047**
Age	−0.02 (−0.03–−0.01)	**0.011**	−0.02 (−0.04–−0.01)	**0.042**
BMI	0.05 (0.03–0.07)	**<0.001**	0.04 (0.02–0.06)	**<0.001**
Duration employment	−0.00 (−0.02–0.01)	0.354	0.01 (−0.01–0.03)	0.203
*Per Capita* Monthly Household Income				
<1999	Reference		Reference	
2000–4,999	−0.04 (−0.30–0.22)	0.744	−0.06 (−0.31–0.19)	0.654
>5,000	−0.27 (−0.77–0.24)	0.304	−0.33 (−0.82–0.16)	0.186
Education level				
Junior middle school and below	Reference		Reference	
High school and above	0.38 (0.22–0.55)	**<0.001**	0.32 (0.15–0.50)	**<0.001**
Smoking				
No	Reference		Reference	
Yes	−0.04 (−0.23–0.14)	0.640	−0.00 (−0.18–0.18)	0.977
Drinking				
No	Reference		Reference	
Yes	0.05 (−0.12–0.22)	0.588	0.03 (−0.14–0.20)	0.710
Exercise				
No	Reference		Reference	
Yes	0.18 (0.01–0.35)	**0.041**	0.09 (−0.08–0.26)	0.288
Marriage				
No	Reference		Reference	
Yes	0.06 (−0.42–0.55)	0.798	0.07 (−0.39–0.54)	0.761

Note: Bolded *p* -values indicate that the association between the variable and HRR level is statistically significant (*p* < 0.05).

Adjusted age, BMI, duration employment, education level, per capita monthly household income, smoking, alcohol consumption, exercise, and marital status. P-Al, plasma aluminum; BMI, Body Mass Index.

**Table 5:** Threshold analysis between plasma aluminum level and HRR.

HRR level	Effect	*p*
Model 1 Fitting model by standard linear regression	−0.17 (−0.34–−0.00)	**0.047**
Model 2 Fitting model by two-piecewise linear regression		
Inflection point	1.68	
<1.68	0.02 (−0.24–0.28)	0.873
≥1.68	−0.56 (−1.15–0.04)	0.067
P for likelihood test		0.194

Note: The bolded *p* value indicates a significant correlation between plasma aluminum levels and HRR level.

Model 1, Model 2 adjusted age, BMI, duration employment, education level, per capita monthly household income, smoking, alcohol consumption, exercise, and marital status.

### 3.4 Relationship between HRR level and total MoCA score

HRR was included as a continuous variable in the multiple linear regression model for statistical analysis, and the results showed that there was a significant positive correlation between HRR and the total MoCA score. Specifically, as the HRR increases, the total MoCA score also increases, i.e., for every unit change in HRR, the total MoCA score increases by 0.45 points on average (Table 6).

**Table 6:** Relationship between HRR level and total MoCA score.

Variables	Single factor		Multifactorial	
	β (95% CI)	*p*	β (95% CI)	*p*
HRR	0.74 (0.39–1.10)	**<0.001**	0.45 (0.09–0.81)	**0.015**
Age	−0.09 (−0.14–−0.05)	**<0.001**	−0.05 (−0.13–0.02)	0.156
BMI	0.07 (−0.00–0.15)	0.051	0.03 (−0.04–0.11)	0.381
Duration employment	−0.07 (−0.10–−0.03)	**<0.001**	−0.01 (−0.07–0.05)	0.782
*Per Capita* Monthly Household Income				
<1999	Reference		Reference	
2000–4,999	0.13 (−0.82–1.08)	0.791	−0.05 (−0.96–0.85)	0.907
>5,000	0.59 (−1.26–2.45)	0.532	0.04 (−1.73–1.81)	0.964
Education level				
Junior middle school and below	Reference		Reference	
High school and above	1.80 (1.19–2.40)	**<0.001**	1.30 (0.66–1.94)	**<0.001**
Smoking				
No	Reference		Reference	
Yes	−0.04 (−0.71–0.63)	0.910	−0.18 (−0.83–0.48)	0.599
Drinking				
No	Reference		Reference	
Yes	0.84 (0.22–1.47)	**0.009**	0.85 (0.24–1.46)	**0.007**
Exercise				
No	Reference		Reference	
Yes	0.65 (0.02–1.28)	**0.044**	0.41 (−0.20–1.03)	0.192
Marriage				
No	Reference		Reference	
Yes	0.02 (−1.76–1.80)	0.982	0.42 (−1.27–2.11)	0.623

Note: Bolded *p* -values indicate that the association between the variable and total MoCA score is statistically significant (*p* < 0.05).

Adjusted age, BMI, duration employment, education level, per capita monthly household income, smoking, alcohol consumption, exercise, and marital status. BMI, Body Mass Index; HRR, hemoglobin-to-red cell distribution width ratio.

### 3.5 Role of HRR in cognitive impairment due to aluminum exposure

**Figure 2:**
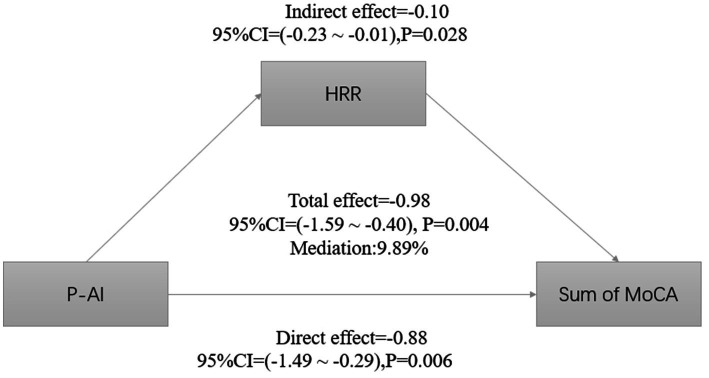
Estimated proportions of correlations between plasma aluminum concentrations and HRR-mediated cognitive scores corrected for age, BMI, length of service, education level, per capita monthly household income, smoking, alcohol consumption, exercise, and marital status.

Since the level of aluminum exposure was significantly correlated with HRR and total MoCA score, and there was also an association between HRR and total MoCA score, we hypothesized that HRR might play a mediating role in cognitive impairment induced by aluminum exposure. We analyzed the mediating role of HRR by using plasma aluminum concentration as the independent variable, cognitive score as the dependent variable, and HRR as the mediator, and the adjustment of covariates was carried out. The results showed that HRR played a partial mediating role between cognitive dysfunction due to aluminum exposure, explaining about 9.89% of the effect (Table 7; Figure 2).

**Table 7:** The coefficients of each path of the mediating effect.

Path	*β*	95% CI	*p*
P-Al → HRR	−0.19	−0.36–−0.03	**0.024**
P-Al → MoCA	−0.90	−1.51–−0.29	**0.004**
HRR → MoCA	0.52	0.17–0.88	**0.004**

Note: Bolded *p*-values indicate that the path effect is statistically significant (*p* < 0.05).

Adjusted age, BMI, duration employment, education level, per capita monthly household income, smoking, alcohol consumption, exercise, and marital status. P-Al, plasma aluminum; HRR, hemoglobin-to-red cell distribution width ratio.

## 4 Discussion

This study explored HRR’s mediating role in aluminum-induced cognitive impairment. Results showed significant links between aluminum exposure and both HRR levels and cognitive dysfunction. HRR independently correlated with cognitive impairment, and mediation analysis indicated that HRR mediated 9.89% of aluminum’s total neurotoxic effect, highlighting its partial role in mediating aluminum’s impact on cognition.

Evidence shows rising human exposure to aluminum, causing health concerns. Aluminum exerts toxic effects on multiple systems, including the nervous, hematopoietic, and skeletal systems, and is associated with encephalopathy, anemia, alumina disease, osteochondrosis and osteoporosis, and other adverse health effects (24). Our previous findings demonstrated that occupational aluminum exposure can induce hematological alterations in workers, which manifests itself early in the form of decreased RBC and Hb levels. The well-documented neurotoxic effects of aluminum accumulation are mediated through proven neurotoxicity, increased inflammation, and oxidative stress (25). This inflammatory microenvironment may disrupt iron metabolism and suppress erythropoietin production, leading to a large number of immature erythrocytes from the bone marrow into the peripheral blood circulation, and the size of erythrocytes involved in the response to peripheral circulation is altered, manifested by an increase in the level of RDW (26). In agreement with our results, aluminum exposure levels are inversely correlated with HRR levels, and the RCS curve further demonstrates the dose–response relationship between plasma aluminum levels and HRR. Supporting these observations, animal studies have reported aluminum-induced erythroid system alterations. Decreased levels of RBC, Hb, and hematocrit and increased mean erythrocyte hemoglobin were observed in these animals (27). Additionally, sub-chronic low-dose aluminum exposure was shown to induce significantly severe dysfunction of the blood, liver, and kidneys in rats (28).

This study further explored the relationship between aluminum exposure and cognitive function. Maintaining metal homeostasis in the central nervous system is crucial for normal brain function, as metals serve as enzyme cofactors and key components in neuronal signaling. Disruption of metal regulation can severely impair neural networks, triggering pathological pathways that lead to oxidative stress, synaptic damage, and cognitive deficits (29). In a study by Campbell et al. that modeled human aluminum exposure through drinking water or diet, researchers observed increased inflammatory activity in mouse brain tissue, including elevated levels of inflammatory cytokines, nitric oxide synthase, and a marker of astrocyte activation, glial fibrillary acidic protein (GFAP). Significantly, Aged rats developed Alzheimer’s disease-like cognitive deficits and neuropathological changes after chronic exposure equivalent to the daily aluminum intake of a portion of the population (30). These findings suggest that chronic low-level aluminum exposure may accelerate a neuron-like aging process, which may lead to the exacerbation of excessive inflammatory responses that in turn drive the course of multiple age-related neurodegenerative diseases.

In the present study, we employed the MoCA scale to evaluate workers’ cognitive performance, as it demonstrates high sensitivity (up to 90%) for early-stage MCI detection (31). Studies have confirmed cognitive impairment associated with environmental aluminum exposure: when analyzed as a continuous variable, each e-fold increase in plasma aluminum concentration was associated with a 0.328-point decreased in total MoCA score; when treated as a categorical variable, plasma aluminum concentration showed a negative correlation with MoCA scores (32); An animal studies provide corroborating evidence, sub-chronic(60-day) low-dose aluminum exposure in Wistar rats through chow and drinking water induced could reach thresholds sufficient to promote memory impairment and neurotoxicity (33). Additionally, shuttle box experiments revealed dose-dependent learning and memory deficits and showed that sub-chronic aluminum exposure impaired learning and memory, cognitive impairment, and hippocampal histological changes are more pronounced with higher aluminum intake (34).

MCI represents a preclinical and transitional stage between normal aging and dementia (35). A meta-analysis of 53 publications found a 15.4% prevalence of MCI in Chinese residents (36). Research shows that long-term Al exposure may lead to mild cognitive impairment (37). With Al being a widespread environmental contaminant, it is crucial to explore the human Al intake threshold sufficient to promote adverse effects. A review related to the harmful effects of aluminum on neurocognition, inflammation, and health described that compared with unexposed workers, workers exposed to occupational conditions experienced adverse changes in neuropsychological tests (involving attention, learning, and memory) (25). A study from Klotz and colleagues presented the well-documented adverse effects of Al on health and peak levels in humans; occupational exposure easily exceeds reference values for maximum internal Al load (<5 μg/L in serum and <15 μg/L in urine) (38). A study on memory impairment in rats found that 60-day subchronic exposure to low doses of Al from feed and added to the water (reflecting human dietary Al intake) reaches a threshold sufficient to promote memory impairment (33). In another cross-sectional study on occupational aluminum exposure populations, it was found that when the plasma aluminum concentration reached 34.52 μmol/L, the cognitive impairment of aluminum-exposed workers mainly occurred in the Digit Span Test, especially in backward digit span (39). Although the current research on the exposure threshold effect of occupational aluminum population is rather complex, it still provides a key direction for the prevention and control of aluminum exposure: clarify the “safety threshold,” formulating aluminum exposure standards; conduct exposure control and use protective equipment; conducting occupational population monitoring to effectively reduce the risks of occupational aluminum exposure.

Detecting cognitive impairment in primary care is challenging due to screening complexities. However, blood-based biomarkers provide accessible, minimally invasive, and cost-effective alternatives, potentially aiding large-scale cognitive screening. Emerging evidence links routine blood parameters based on routine blood and biochemical tests, including Hb, platelets, and physiologic ion concentrations, are associated with cognitive impairment (40). Specifically, reduced Hb levels have been associated with cognitive decline, which may be attributed to decreased cerebral hypoxia or aerobic capacity due to low Hb levels (41); and community-based research has further established associations between increased RDW and impairment in all cognitive domains of MCI (42). Notably, increased RDW has been implicated in the inflammatory pathogenesis of AD (43).

The hemoglobin-to-red blood cell distribution width ratio (HRR) is recognized as a marker of inflammation associated with the incidence of various diseases and adverse events, and is an indicator of the level of inflammation and immunometabolism in the body. Neuroinflammation is key in neurodegenerative diseases. To facilitate early detection of dementia-related pathological changes as early as possible, considerable biomarker research has increasingly focused on several markers of peripheral inflammation that are key elements of neurodegenerative changes, including cytokines, lymphocytes, neutrophils, and various blood cell indices (10). These inflammatory indices offer practical advantages of being highly reproducible and low cost, and they have been widely used in a variety of neurodegenerative diseases (11, 44, 45). The HRR provides a sensitive measure of red blood cell function, and this ratio tends to rise as inflammation improves (16). Notably, low HRR levels have been identified as a low level of HRR could be an independent risk factor (14). For instance, in chronic obstructive pulmonary disease (COPD) patients has shown that HRR has some predictive value for mortality in COPD participants, and can be used as a simple and convenient tool to identify high-risk patients and guide targeted interventions, which may contribute to a certain extent to the reduction of mortality in patients with COPD (46). Higher HRR levels correlate with lower stroke risk, highlighting its importance in identifying stroke-prone patients (16). Despite extensive investigations into aluminum exposure and the relationship between aluminum exposure and cognitive function, there are still some notable absences regarding the specific role and impact of HRR in this regard. Thus, HRR, reflecting inflammation, may serve as a potential marker for early cognitive impairment detection.

Our findings demonstrate that the hemoglobin-to-red cell distribution width ratio (HRR) mediated approximately 9.89% of the cognitive impairment induced by aluminum exposure. Although this mediating effect does not represent the dominant pathway, its statistical significance suggests HRR may constitute one potential mechanism underlying aluminum-induced neurotoxicity. The level of HRR reflects the anemia and inflammatory state of the organism, chronic and acute cerebral hypoxia may exist as a result of respiratory, cardiovascular, or anemia disorders, and hypoxia is widely associated with cognitive deficits (47). Recent studies have shown that a low Hb/RDW ratio is associated with an increased risk of cognitive impairment and dementia. This might be attributed to the dual effects of low hemoglobin (leading to insufficient oxygenation in the brain) and high RDW (indicating potential underlying pathology and inflammation) (48). Neuroinflammation, a well-documented driver of neurodegeneration and AD progression (49).

The advantages of this study are as follows: this study is the first cross-sectional research on the association between HRR and cognitive function in the occupational aluminum exposure population, the focus was placed on the occupational aluminum exposure population, filling the evidence gap in the association between HRR and cognitive function in this special exposure population. Combining the traditional anemia differentiation indicator of the ratio of hemoglobin to red blood cell distribution width and extending the HRR indicator to the field of cognitive function research, a new perspective has been proposed for its potential biomarker of early neurological dysfunction. Through a cross-sectional study design, a direct association between HRR and cognitive function can be rapidly established, providing a hypothesis basis for subsequent longitudinal mechanism research. These findings highlight HRR’s dual value as both an early warning indicator and intervention target: If elevated HRR is primarily driven by anemia, correction of anemia may partially reverse cognitive impairment, and HRR could be incorporated into multi-targeted intervention (such as concurrent control of aluminum exposure, improvement of anemia, antioxidant therapy) rather than as a stand-alone target of intervention. Targeted interventions may be of more pronounced benefit in aluminum-exposed individuals with abnormal HRR (such as anemia combined with high RDW). This study provides simple and economical blood routine-derived indicators for the early screening of neurotoxicity among aluminum workers and optimizes occupational health monitoring strategies.

However, this study has certain limitations: firstly, the moderate sample size may limit the generalizability of our findings, so the results should be treated with caution; secondly, this study is a cross-sectional design, so it is not possible to clarify the causal relationship between plasma aluminum exposure levels, HRR levels, and cognitive impairment. Thirdly, the subjective/anamnestic data of this study were mainly used to establish inclusion/exclusion criteria, ensuring the homogeneity of the cohort and minimizing confounding effects to the greatest extent. Although this method enhanced internal effectiveness, it failed to capture the subjective complaints reported by the research subjects. Fourth, this study did not measure the aluminum concentration in the external working environment where aluminum workers were located, and lacked specific environmental aluminum concentration data, making it difficult to determine the specific level of aluminum exposure of workers and their direct association with health effects. This also limits our ability to fully understand the potential risks to worker health from aluminum exposure in the external work environment. Finally, current research reveals the correlation characteristics among variables through statistical models. However, the underlying biological regulatory mechanisms have not yet been verified through experimental means. The biological rationality of this approach still requires further clarification through subsequent studies in combination with molecular biology, neurophysiology, or neuroimaging.

Our research results indicate that the level of aluminum exposure is significantly negatively correlated with the total MoCA score: the higher the aluminum exposure, the lower the total MoCA score. It was also significantly negatively correlated with HRR, and this association remained significant even after controlling for related variables. In addition, HRR is significantly positively correlated with the total score of MoCA. This advances our understanding of aluminum toxicity mechanisms and how environmental factors impact brain health via blood components. Clinically, monitoring HRR in aluminum-exposed workers can identify changes due to high aluminum exposure and reduce cognitive impairment. Therefore, future studies should expand the sample size, consider a longitudinal design to more accurately assess the dynamic role of HRR between aluminum exposure and cognitive impairment, and incorporate detailed environmental monitoring data to more accurately quantify the level of aluminum exposure and to explore its relationship with health effects. Given that this study focuses on the health monitoring of the occupational aluminum exposure population and aims to rapidly assess the inflammatory status of the group through the basic data in routine blood tests, the selection of this indicator does not deny the clinical value of classic inflammatory indicators such as C-reactive protein (CRP), PCT, or WBC. Rather, it is a targeted choice made based on the actual demands for the convenience, economy, and operability of detection in large-scale occupational population screening. Future research can further incorporate these indicators. Through the joint analysis of multiple indicators, the characteristics of inflammatory responses related to occupational aluminum exposure can be revealed more comprehensively.

## 5 Conclusion

Occupational aluminum exposure increases the risk of HRR levels and cognitive impairment. HRR levels may be a mediator of cognitive impairment due to aluminum exposure. It is recommended that occupational aluminum workers focus on routine blood markers in their daily physical examinations and have their HRR levels monitored regularly.

## Data Availability

The original contributions presented in the study are included in the article/supplementary material, further inquiries can be directed to the corresponding author.
